# The World’s Columbian Dental Congress

**Published:** 1892-09

**Authors:** 


### The World's Columbian Dental Congress.

The General Executive Committee of the World's Dental Congress met at Lookout Mountain, Tenn., Monday, July 25th. All except three members present. The committee was busily engaged for about two days arranging matters for the great congress. Some additional committees were appointed. An important part of the work accomplished here was specifying the duties of the various committees, so that each may understand clearly the work it has before it. A large number of persons was appointed, not only to the new committees but upon committees that had already been appointed. It is evident that if the work in any particular fails it will not be for want of a goodly number of persons on each committee.

The Finance Committee, one of the most important of the whole list of committees, had not found its way for work open and clear till this meeting; its chairman and the other members of its committee have now taken up the work in earnest. To carry the work of the congress in all its details and to keep it fully abreast with the other departments of the great Exposition will rquire a large amount of money, probably from fifty to seventy-five thousand dollars, and in order that there may be no lack in this respect we suggest that the members of the profession to whom the Finance Committee may apply respond liberally and promptly ; no one should hesitate for a moment to make a liberal contribution. This will be the grandest event for dentistry the world has ever seen, one in the results of which every dentist will be personally interested, and in a variety of ways it will add honor, dignity and importance to the profession if it is properly carried through. No work done by the committee on the occasion of its recent meeting was of more importance, perhaps, than the consideration of the finance question.

A great deal of other work was done by the committee, and the utmost harmony and warm interest, even to enthusiasm, seemed to pervade the mind of every member of the committee.

A circular of information will very soon be issued by the Editorial Committee which will indicate the present status of the enterprise.

The next meeting of the Executive Committee will be held in Cincinnati on Monday, October 24th, when it is to be hoped that every member of the committee will be present.

